# Abdominal Flap Necrosis and Wound Dehiscence following a Medical Tourist Tummy Tuck

**DOI:** 10.1155/2020/8819102

**Published:** 2020-11-24

**Authors:** Vladislav Pavlovich Zhitny, Noama Iftekhar, Peter Caravella, Jake Patrick Young, Barry Zide, Frank Stile

**Affiliations:** ^1^School of Medicine, University of Nevada, Las Vegas, Las Vegas, NV, USA; ^2^School of Medicine, Loyola University of Chicago, Maywood, IL, USA; ^3^Las Vegas Surgical Associates, Las Vegas, NV, USA; ^4^Stile Aesthetics, Las Vegas, NV, USA; ^5^New York University, Langone Health, New York City, NY, USA

## Abstract

Abdominoplasty is a major surgical procedure met with high rates of patient satisfaction and improved self-image. While many patients are lured abroad due to discounted prices for such highly requested procedures, unfortunately, there are also associated complications. A 47-year-old woman presented due to abdominal scar dehiscence due to skin necrosis secondary to a discounted abdominoplasty in Mexico. The patient had been turned away by several local surgical centers for treatment of the necrosis. The patient underwent incision, drainage, and two debridements before her abdominal wound was eventually closed. Patient recovered well postoperatively with improved aesthetic result. With the rise of social media advertisements, more patients elect to receive plastic surgery abroad. Unfortunately, many of these practices are not accurately vetted and this can complicate the postoperative care especially upon return to the United States.

## 1. Introduction

Abdominoplasty is the fifth most common plastic surgery procedure performed in the United States [1, 2]. The procedure corrects weakness of the abdominal musculature and redundant lower abdominal skin. It is associated with high patient satisfaction rates and improved self-image, even in the face of complications.

As a major surgery, a number of complications can arise [2, 3]. Skin necrosis, a rare complication postoperatively, results in abdominoplasty flap dehiscence which can present with decreased temperature of the area and slow capillary refill [3–5]. The sequelae are often due to insufficient perfusion secondary to blood flow interruption, tight garments, or pathology associated with poor wound healing like diabetes or lupus. It is important for a surgeon to have a good understanding of the abdominal vasculature to ensure the skin flaps of the abdominoplasty remain well-perfused.

Studies have found that in patients who suffered complications following completion of cosmetic procedures abroad, abdominoplasty yielded the highest rate of complications [6, 7]. Medical tourists, as they are called, frequently visit countries, such as Mexico, the Dominican Republic, and the Philippines, hoping to complete cosmetic enhancements at a fraction of the cost charged by U.S. surgeons.

Many times, these patients follow a recommendation they receive from a friend or see an online solicitation. These patients often lack the necessary expertise when searching for a prospective foreign surgical facility that meets the proper standard of care. The resulting complications from such procedures pose a significant burden to the public health care system. The reported cost of correcting these misadventures can range from $4,553 in minor cases and up to $55,569.66 for patients requiring complex corrections [6, 7].

In this paper, we wish to present a case of a medical tourist who suffered abdominal skin flap necrosis following abdominoplasty performed in Mexico. The healing and corrective interventions following initial skin necrosis took two months to complete and proved to be quite challenging emotionally, physically, and financially for the patient.

## 2. Case Presentation

**Figure 1:**
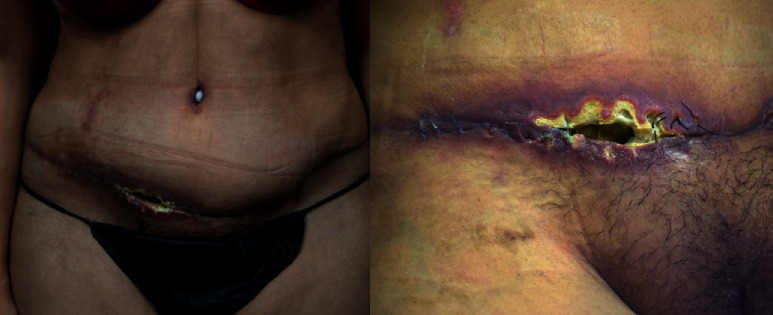
The patient's ventral view with initial dehiscence and flap necrosis.

A 47-year-old female presented for evaluation to the clinic for an abdominal scar dehiscence secondary to a failed abdominoplasty procedure (Figure 1). In 2019, the patient had traveled to Mexico seeking a low-cost abdominoplasty. The patient reported that she felt her incision became “infected” and that the sutures were “unraveling.” The initial procedure was performed approximately 2 weeks prior to our initial evaluation. Before presenting to our practice, the patient was refused evaluation and/or treatment by several plastic surgeons, not uncommon for patients who develop complications from elective cosmetic surgery procedures performed abroad.

The patient was in obvious distress, and when inquired if she wanted to return to the operating surgeon in Mexico, she declined. Prior to the surgical procedure, the patient weighed 58.8 kg and her height was 1.54 m (body mass index: 24.1 kg/m^2^). Patient's preoperative labs and vitals were normal. She was in overall good health and cleared for debridement surgery followed by subsequent surgery for scar closure.

The lower right lateral abdomen showed necrosis of the skin flaps and underlying tissue measuring 8 centimeters in length and 5 centimeters in width in its greatest dimensions.

### 2.1. Operative Procedure

**Figure 2:**
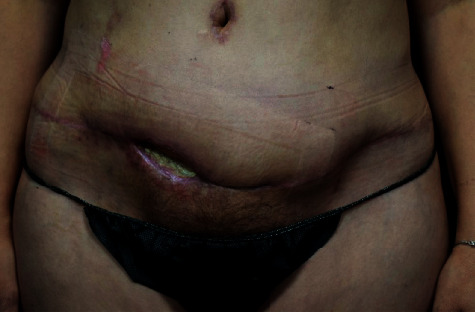
The patient's ventral view with healing contracted wound following initial debridement.

After obtaining consent for an incision, drainage, and debridement, the patient was brought to the operating room under general anesthesia. The nonviable tissue and skin edges were debrided using sharp dissection with a scalpel and the electrocautery. The wound was washed out and packed with normal saline-soaked Kerlix. This was done in anticipation of providing this patient with regular focused wound care. Our intention was to provide wet-to-dry wound debridement while allowing the wound to begin healing through secondary intention (Figure 2).

**Figure 3:**
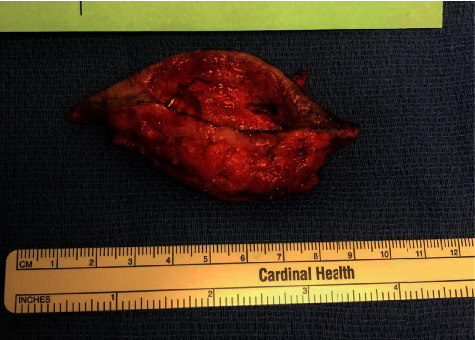
Excised abdominal wound and skin edges (7.75 cm).

One month from the debridement procedure, the patient returned for second debridement and closure of her abdominal wound (Figure 3). After undermining the abdominal flap superiorly, the abdominal and groin skin edges were easily approximated without tension over a number 7 Jackson-Pratt drain.

### 2.2. Results: Follow-Up

**Figure 4:**
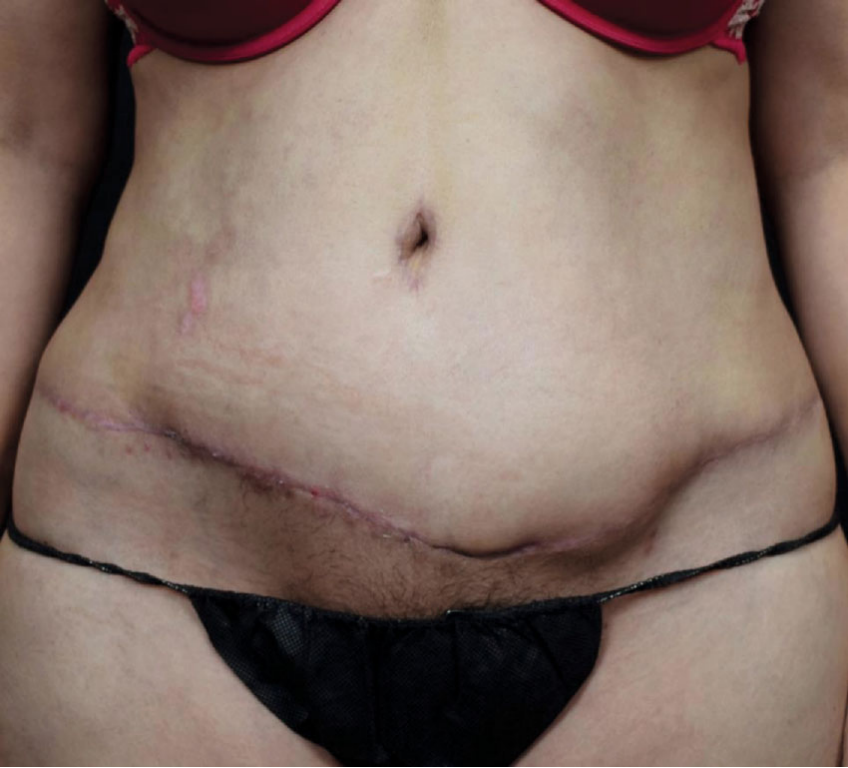
The patient's ventral view following wound debridement and closure.

Patient recovered well with no complications following completion of her procedures. At post-op day 30, the patient's wound demonstrated good healing (Figure 4).

## 3. Discussion

Plastic surgery abroad and medical tourism have grown in popularity over the last decade, especially with growing acceptance for cosmetic procedures. The price for procedures performed in the United States proves to be cost prohibitive for many individuals seeking cosmetic enhancement. A survey conducted in 2008 demonstrated that 39% of Americans would seek elective plastic surgery abroad if they could pay half of its cost [8]. However, seeking cosmetic surgery out of the United States proves to be a risky endeavor. Flying soon after completion of the procedure is associated with an increased risk of deep venous thrombosis [9]. Currently, few studies exist on the rates of complication associated with medical tourism, but a survey completed by the British association of Plastic, Reconstructive, and Aesthetic Surgery found that 37% of plastic surgeons had patients present with complications related to seeking cosmetic surgery abroad. Another study completed by Oman surveyed that 15% of 45 medical tourists had complications [10–12]. Comparatively, a study in the United States which examined 26,032 consecutive surgeries (including breast augmentation, liposuction, and facial cosmetic surgery) found a 0.78% rate of complication [13]. Among the many open problems following procedures performed on medical tourism, or out of necessity while abroad, is the underestimation of thromboembolic risk. Patients and medical practitioners should have a better understanding of such medical postoperative complication, and management should be in keeping with the latest international guidelines [14]. Legal protections, which safeguard practices and surgeries in America, do not extend abroad [15, 16]. These include FDA- (Food and Drug Administration-) approved products, including implants deemed safe for the public.

While there are many qualified plastic surgeons abroad, patients seeking a cheap alternative often fail to conduct appropriate background research. Electing to have cosmetic surgery abroad loses valuable follow-up visits that take place at weekly and monthly intervals postoperatively. Those who require follow-up care and revisions may face difficulties transferring care to a local surgeon, who is likely unaware of the details behind the original procedure. This can lead to complex, costly revisions.

Current recommendations for care include hyperbaric oxygen and negative-pressure wound therapy for wound closure. For our patient, we used initial sharp debridement, focused wound care, and secondary wound closure as treatment. The healing process following some skin necrosis cases can take up to eight months, which can prove to be agonizing for the patient. Complications following cosmetic surgery can be particularly unsettling because of the expectations of an “enhancement” or “appealing” final result.

## 4. Conclusion

We recommend for patients to seek care in the United States or at a facility that is associated with a clinic in the United States for postoperative care. In the case of complications or poor results, patients should seek a plastic surgeon for assistance.
